# CT-based subchondral bone microstructural analysis in knee osteoarthritis via MR-guided distillation learning

**DOI:** 10.3389/fradi.2026.1798348

**Published:** 2026-04-10

**Authors:** Yuqi Hu, Xiangyu Zhao, Gaowei Qing, Kai Xie, Chenglei Liu, Lichi Zhang

**Affiliations:** 1 School of Biomedical Engineering, Shanghai Jiao Tong University, Shanghai, China; 2 Department of Radiology, Shanghai Ninth People's Hospital, Shanghai, China; 3 Shanghai Key Laboratory of Orthopaedic Implants, Department of Orthopaedic Surgery, Shanghai Ninth People’s Hospital, Shanghai, China

**Keywords:** cross-modal registration, distillation learning, knee osteoarthritis, subchondral bone analysis, computed tomography

## Abstract

Reliable analysis of subchondral trabecular microstructure is critical for knee osteoarthritis assessment. However, this analysis largely relies on high-resolution MRI acquired using balanced fast field echo (BFFE) sequences, which are rarely included in routine clinical protocols. Clinical CT is widely acquired, yet its limited spatial resolution and soft-tissue contrast makes direct trabecular parameter estimation unreliable. Therefore, it is specifically demanded to enable accurate trabecular microstructural analysis and osteoarthritis diagnosis using routine clinical CT, while also approaching the reliability of MR-based analysis. In this paper, we propose CT-based Subchondral Microstructural Analysis (CT-SMA) method, which utilizes distillation learning technology to transfer high-resolution structural knowledge from MR to CT while enforcing CT-only inference. The core idea of CT-SMA is to transfer microstructural knowledge learned from high-resolution MR to CT through cross-modal knowledge distillation, using a pre-trained MR-based teacher model to supervise CT-based student model on feature maps. To support effective distillation, CT-SMA further introduces a synthesis-based, multi-stage MR–CT registration strategy that establishes patch-level correspondences across modalities, despite substantial differences in resolution, contrast, and appearance. Experiments on a clinical knee imaging cohort demonstrate that CT-SMA substantially improves CT-based trabecular parameter estimation, achieving strong agreement (ICC = 0.742) with MR-derived references across key trabecular biomarkers. Moreover, when aggregated using a Transformer-based model, the regressed CT-derived parameters enable patient-level osteoarthritis diagnosis with an AUC of 0.883, substantially outperforming CT-based prediction without distillation (AUC = 0.778). These results indicate that routine clinical CT can support reliable subchondral bone analysis via proposed CT-SMA, establishing a practical foundation for large-scale studies.

## 1 Introduction

Knee Osteoarthritis (KOA) is a prevalent degenerative joint disease that causes pain and disability in elder individuals (1, 2). Accumulating evidence suggests that microstructural alterations in subchondral trabecular bone are closely linked to early disease onset and progression, often preceding visible cartilage degeneration (3). Quantitative trabecular biomarkers such as bone volume fraction (BV/TV), trabecular thickness (Tb.Th), trabecular separation (Tb.Sp), and trabecular number (Tb.N) have been shown to correlate with histological degeneration, biomechanical competence, and long-term structural outcomes (4, 5). High-resolution MRI acquired with balanced fast field echo (BFFE) sequences, provides the spatial detail required to compute these biomarkers reliably (6). However, its long acquisition time, high cost and limited accessibility restrict large-scale use in clinical environments and population studies (7). On the other hand, routine clinical CT is widely available and fast to acquire, but its voxel resolution (150–500 μm) is substantially coarser than trabecular bone microstructure (50–200 μm) (8, 9). This substantial resolution mismatch leads to strong partial volume effects and makes trabecular morphology almost indiscernible on CT images (10, 11). As a result, recovering MR-level trabecular information from routine CT remains technically challenging, despite its potential to enable scalable and cost-effective assessment of subchondral bone microstructure in osteoarthritis.

To enable reliable subchondral trabecular microstructural analysis using clinical CT, the fundamental challenge lies in compensating for the severe loss of fine-scale structural information inherent to CT acquisition. This information loss makes it difficult for traditional radiomics analysis approaches to extract stable microstructural descriptors, and limits the effectiveness of deep learning models that attempt to regress trabecular parameters directly from CT appearance. In contrast, high-resolution MR provides detailed representations of trabecular architecture and the relationship between MR texture patterns and quantitative biomarkers is well established (12). In this way, the anatomical structure information along with the trabecular parameters obtained from the acquired MR images can serve as an explicit supervisory signal to guide parameter estimation from CT.

The technology of cross-modality knowledge distillation (13, 14) provides a natural mechanism for transferring MR-based structural knowledge to optimize CT-based model, and resolve the issues in CT-based subchondral bone microstructural analysis. In practice, for paired MR/CT patches from the same anatomical region, a prediction-consistency loss is used to distill trabecular-parameter knowledge from the MR-based teacher model to the CT-based student model. However, successful distillation process requires CT patches to correspond accurately to their MR counterparts, so that the MR model can act as an effective teacher during training. Therefore, it is demanded to establish accurate MR-CT registration to make reliable correspondence between the MR and CT patches. Nevertheless, such cross-modal registration is challenging due to their considerable resolution difference, their substantial contrast and appearance discrepancy, and the frequent variation in knee flexion angle between CT and MR acquisitions.

To address these challenges, we propose a novel CT-based Subchondral Microstructural Analysis (CT-SMA) method, which is a unified computational framework designed to enable reliable trabecular analysis and osteoarthritis assessment using routine clinical CT. The central idea of CT-SMA is to leverage high-resolution MR as a teacher modality during training, while operating exclusively on CT at inference time. To achieve this, CT-SMA integrates three tightly coupled components: **First**, to make distillation feasible in practice, we introduce a synthesis-guided MR–CT registration module that establishes anatomically faithful cross-modal patch correspondences despite large contrast/resolution gaps and pose variations. **Second**, built on these aligned MR/CT patch pairs, we develop a distillation-based regression module (DLRM) that transfers MR-derived trabecular-parameter knowledge to a CT model via a prediction-consistency objective. **Finally**, to support patient-level OA assessment, we design a transformer-based aggregation module that integrates spatially distributed trabecular parameters into a global representation for downstream prediction. We demonstrate that this strategy substantially improves the reliability of CT-based trabecular parameter estimation and enables downstream OA analysis with performance approaching MR-based methods. The main contributions of this work are summarized as follows:

(1) We present CT-SMA, a unified framework that bridges MR-guided training and CT-only inference for subchondral trabecular analysis.
(2) We propose a synthesis-guided cross-modal registration pipeline that produces anatomically consistent MR–CT patch correspondences for distillation.
(3) We introduce a cross-modality distillation strategy to transfer high-resolution MR structural priors to CT for accurate trabecular-parameter regression.
(4) We validate the clinical utility of the distilled CT representations on OA prediction, achieving large gains over CT-only baselines and nearing MR-based performance.

## 2 Related work

### 2.1 Trabecular microstructure analysis for OA classification

Trabecular microstructure has been increasingly recognized as a prognostic substrate for osteoarthritis (OA), motivating computational methods that quantify trabecular biomarkers from clinical imaging and leverage them for OA prediction. Early studies on radiographs primarily relied on classical trabecular texture analysis. They used statistical descriptors of local intensity patterns, and demonstrated that subchondral trabecular patterns are predictive of OA status and progression. However, radiographs provide only two-dimensional projections and typically rely on handcrafted features that are sensitive to acquisition conditions and offer limited structural fidelity (15, 16). In contrast, high-resolution magnetic resonance imaging has enabled more direct characterization of trabecular microarchitecture. In particular, balanced fast field echo (BFFE) sequences have been widely adopted for subchondral bone microstructural analysis of the knee region (2, 17). By exploiting steady-state free precession and strong T2/T1 contrast, BFFE provides high spatial resolution and clear delineation between bone, cartilage, and marrow spaces, allowing individual trabeculae to be resolved more reliably than conventional sequences. Based on BFFE acquisitions, classical three-dimensional morphometric parameters such as bone volume fraction (BV/TV), trabecular thickness (Tb.Th), trabecular separation (Tb.Sp), and trabecular number (Tb.N) can be estimated, and these biomarkers have shown strong associations with OA severity, progression, and cartilage degeneration (3). Reported results show strong associations between MR-derived features and the underlying trabecular structure (3), yet its clinical application has been undermined due to the high acquisition cost (7).

Conversely, CT-based trabecular analysis has also been explored, but mainly in controlled or *ex vivo* settings where scan quality is sufficiently high to extract three-dimensional structural descriptors linked to biomechanical properties. These studies suggest that CT-derived structural features are informative for osteoarthritic bone changes, but this evidence is largely obtained under high-resolution imaging conditions that are uncommon in clinical CT (18, 19). Collectively, prior work establishes the predictive value of trabecular microstructure for OA, yet existing approaches either require high-resolution imaging that do not address the core challenge of recovering fine-scale trabecular information from routine low-resolution clinical CT.

### 2.2 Cross-modal image registration

Cross modality registration plays a fundamental role in medical image analysis as it enables the integration of complementary anatomical information across imaging modalities for tasks such as diagnosis, treatment planning and multimodal learning. However, multimodal alignment remains challenging due to substantial appearance discrepancies between modalities, complex geometric inconsistencies introduced by patient positioning, and the instability of nonconvex optimization procedures required for deformable matching. Traditional approaches rely either on intensity-based similarity measures or on deformable transformation models, but these methods often struggle to obtain reliable correspondences when appearance differences are large (20). More recent learning-based registration techniques focus primarily on unsupervised prediction of deformation fields, yet their reliance on intensity-based consistency limits their robustness in cross modality settings (21). Synthesis-assisted registration attempts to reduce the appearance gap by generating modality converted images before alignment, although synthesis errors and structural distortions frequently impair the accuracy of the downstream registration (20, 22).

Knee-specific MR and CT registration has also been investigated using segmentation-based workflows that rely on bone surface extraction followed by intensity driven alignment. Prior work demonstrated that registering MR and CT bone surfaces is inherently challenging because MR provides limited visibility of cortical boundaries, which reduces the reliability of surface-based correspondence and highlights the difficulty of establishing accurate anatomical alignment between the two modalities. More advanced strategies register individually-segmented skeletal components as rigid bodies and interpolate the surrounding deformation field before applying a rigidity constrained deformable refinement (23). Although these approaches improve geometric alignment of bone structures, they depend on accurate bone segmentation and primarily operate on surface representations rather than volumetric correspondences. Overall, existing studies in this area remain limited, and learning-based multimodal registration methods for the knee are still relatively underexplored, particularly in scenarios that require voxel level alignment within trabecular bone for downstream microstructural analysis (24).

### 2.3 Cross-modal knowledge distillation

Knowledge distillation provides a mechanism for transferring useful information between models or modalities by encouraging consistency at the prediction or feature level, enabling complementary modality specific information to be shared across representations (25). Cross modal distillation techniques typically operate through divergence minimization between output distributions, consistency regularization on intermediate features, or adversarial learning schemes that promote modality invariant embeddings (26).

In medical image analysis, cross modality distillation has been studied primarily in the context of segmentation. One representative approach integrates an image alignment module with online mutual distillation to exploit modality shared shape priors and enhance segmentation performance across CT and MR modalities (13, 26). Another line of work achieves unpaired multimodal segmentation by parameter sharing and constraining the prediction distributions of CT and MRI through a distillation inspired loss, significantly improving segmentation accuracy with a compact model architecture (27). Beyond segmentation, cross modal distillation has been used to address incomplete multimodal data in neuroimaging, where a multimodal model serves as a teacher to guide a single modality student for Alzheimer's Disease classification (28).

Although these approaches demonstrate the effectiveness of cross modal distillation for classification and segmentation, they primarily focus on semantic outputs and do not address continuous microstructural biomarker regression, large resolution discrepancies between modalities, or the need for patch level anatomical correspondence within trabecular bone. To the best of our knowledge, cross modal distillation has not been explored for knee osteoarthritis analysis or subchondral trabecular microstructure estimation.

## 3 Method

**Figure 1:**
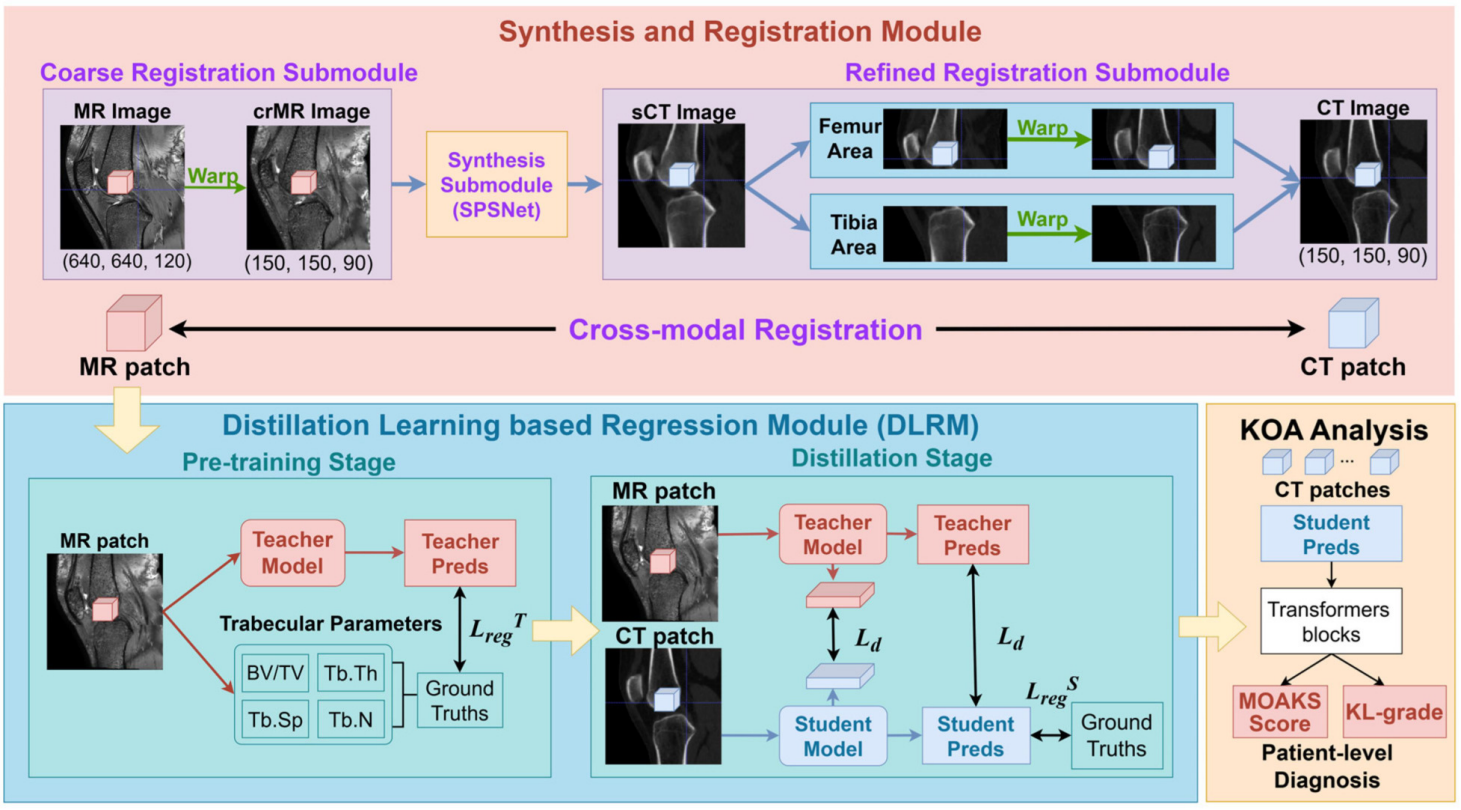
Our CT-SMA framework for cross-modal trabecular parameter regression and subchondral-bone-microstructural-analysis. The synthesis and registration module achieves MR-to-CT alignment through three dedicated submodules. First, a coarse MR-to-CT registration submodule aligns MR images to CT space to generate crMR images. Second, a synthesis submodule based on SPSNet transforms crMR images into sCT while preserving anatomical structures. Third, a refined registration submodule performs component-wise registration from sCT to CT for individual bone regions. With paired MR and CT patches, the distillation-learning-based regression (DLRM) module first pre-trains an MR-based teacher model and then supervises the CT-based student model with knowledge distillation. The regressed parameters are further employed for KOA analysis, including KL-grade-based KOA classification and MOAKS score prediction.

**Figure 2:**
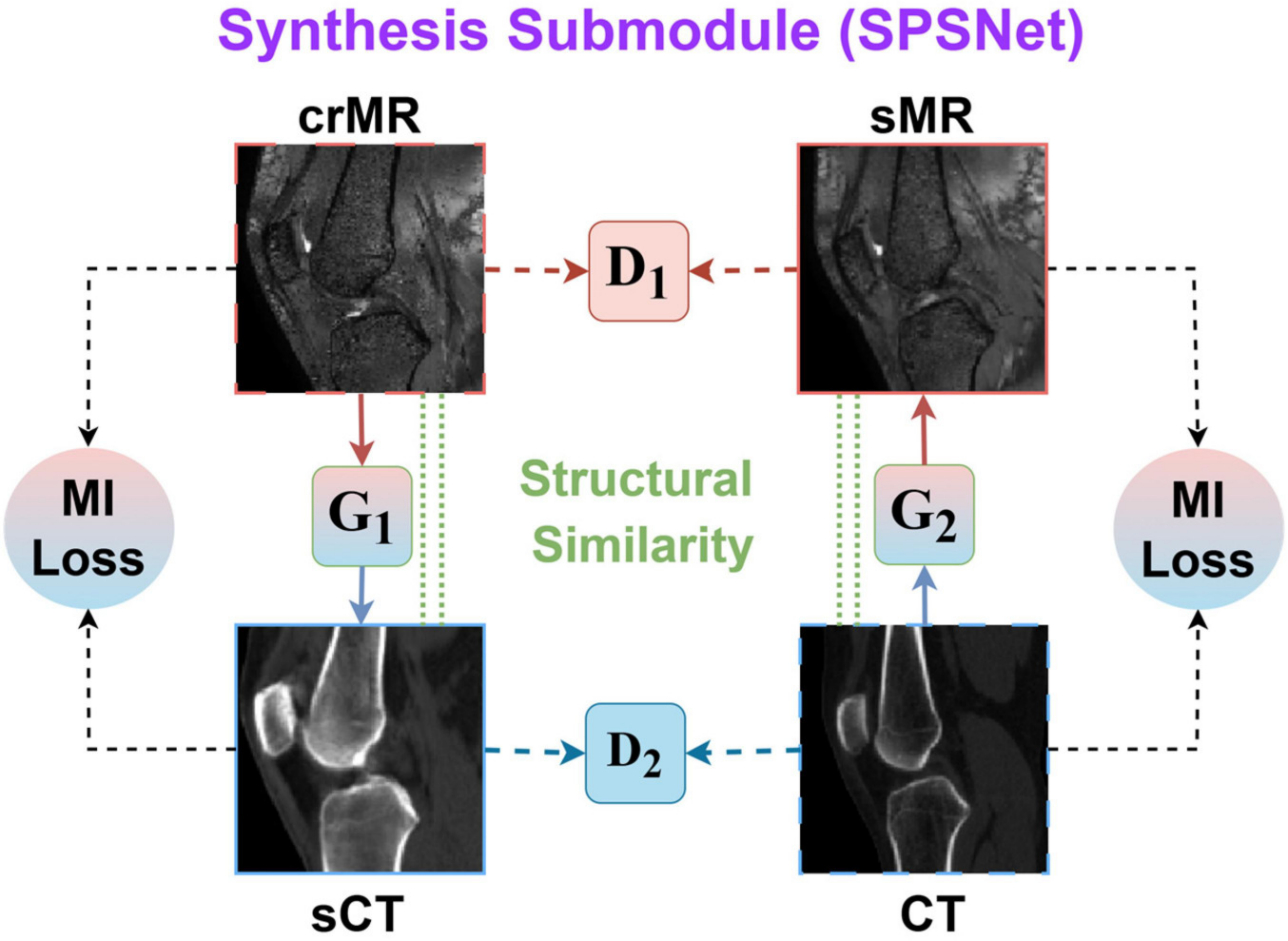
Detailed designs of submodules in CT-SMA. **(A)** Shows the structure of SPSNet, which consists of two generators ($G_{1}$ and $G_{2}$) and two discriminators ($D_{1}$ and $D_{2}$). The mutual information (MI) loss constrains the structural similarity during cross-modal synthesis process.

The overall pipeline of CT-SMA method is illustrated in Figure 1, which aims to enable trabecular parameter regression from clinical CT by transferring high-resolution structural information from MR. Let $I^{MR}$ and $I^{CT}$ denote the original MR and CT volumes of size H × W × D. As shown in Figure 2, CT-SMA contains three major components. The first component constructs anatomically meaningful correspondences between modalities through synthesis and registration. The second component performs patch-level distillation learning to transfer trabecular knowledge from MR to CT. The final component aggregates patch-level predictions into a patient-level representation for osteoarthritis classification.

### 3.1 Synthesis and registration module

CT and MR exhibit substantial discrepancies in field of view, voxel spacing, intensity distribution, and knee articulation. Direct voxel-level alignment is unreliable due to these differences. Therefore, this module progressively reduces cross-modal discrepancies through three stages: coarse global alignment, structure-preserving synthesis and bone-wise refined alignment.

#### 3.1.1 Coarse registration

The goal of coarse registration is to eliminate global misalignment in field of view and resolution. We estimate a rigid transformation $R_{g}$ that maximizes mutual information between the transformed MR volume and CT:

$$R_{g}=\arg\underset{R}{\max}\;\mathrm{MI}(R(I^{MR}),I^{CT}).$$

Mutual information is defined as

$$MI(A,B)=\underset{a}{\sum }\;\underset{b}{\sum }\;p_{AB}(a,b)\log\frac{\;p_{AB}(a,b)}{\;p_{A}(a)p_{B}(b)},$$

which provides robustness for multimodal alignment. Applying the rigid transform yields the coarsely aligned MR volume as

$$I^{crMR}=R_{g}(I^{MR}).$$

This volume is now spatially consistent with CT, providing the foundation for subsequent finer alignment.

#### 3.1.2 Structural-preserving synthesis submodule (SPSNet)

Although coarse registration reduces global spatial misalignment, direct multimodal registration remains challenging due to the large intensity distribution gap between MR and CT. To mitigate this issue while preserving anatomical structures, we design a Structural Preserving Synthesis Network (SPSNet). As illustrated in Figure 2, the key idea is to map MR to a CT-like appearance and map CT to an MR-like appearance, while enforcing structural consistency using mutual information.

Let $I^{crMR}$ denote the coarsely registered MR volume and $I^{CT}$ denote the CT volume. SPSNet contains two generators: The generator $G_{1}$ synthesizes a CT-like image from MR, and $G_{2}$ synthesizes an MR-like image from CT:

$$\begin{array}{c} I^{sCT}=G_{1}(I^{crMR}) \end{array},\begin{array}{c} I^{sMR}=\;G_{2}(I^{CT}) \end{array}.$$

Two discriminators $D_{1}$ and $D_{2}$ are used to distinguish synthesized images from real images in the corresponding target domains. The adversarial objectives are defined as:

$$L_{\mathrm{GAN}}^{CT}=E_{I^{CT}}[\log\;D_{2}(I^{CT})]+E_{I^{crMR}}[\log(1-D_{2}(G_{1}(I^{crMR})))],$$

$$L_{\mathrm{GAN}}^{MR}=E_{I^{crMR}}[\log\;D_{1}(I^{crMR})]+E_{I^{CT}}[\log(1-D_{1}(G_{2}(I^{CT})))].$$

To prevent structural distortions and ensure content preservation during synthesis, the cycle consistency loss is computed as:

$$L_{\mathrm{cyc}}=\|G_{2}(I^{sCT})-{I^{crMR}|}_{1}\|+\|G_{1}(I^{sMR})-I^{CT}{\|}_{1}.$$

To encourages preservation of anatomical structures, we explicitly enforce structural similarity between the synthesized images and their corresponding inputs using mutual information. Specifically, we impose mutual information consistency between the coarsely registered MR image and its synthesized CT counterpart, as well as between the original CT image and the synthesized MR image:

$$L_{\mathrm{MI}}=-\mathrm{MI}(I^{crMR},I^{sCT})-\mathrm{MI}(I^{CT},I^{sMR}).$$

By maximizing mutual information across synthesis directions, SPSNet discourages geometric distortions and hallucinated structures while allowing flexible intensity translation across modalities.

The overall objective of the synthesis submodule is defined as the weighted sum of adversarial, cycle consistency, and mutual information losses:

$$L_{\mathrm{SPS}}=L_{\mathrm{GAN}}^{\mathrm{CT}}+L_{\mathrm{GAN}}^{\mathrm{MR}}+\lambda_{cyc}L_{\mathrm{cyc}}+\lambda_{MI}L_{\mathrm{MI}}.$$

This step produces a CT-like representation that remains structurally faithful to MR and enables robust mono-modal alignment in the next stage. The weighting coefficients were set to $\lambda_{cyc}=1.0$ and $\lambda_{MI}=0.5$. A larger weight is assigned to the cycle-consistency loss to preserve semantic consistency during cross-modal translation, while the mutual information term provides an auxiliary constraint to encourage anatomical structure consistency across modalities.

#### 3.1.3 Refined bone-wise registration

After synthesis, residual misalignment persists due to differences in knee-bent angles during MR and CT acquisition. Importantly, femur and tibia exhibit distinct rigid motions that cannot be adequately modeled by a single global transformation. To address this articulation-dependent misalignment, we design refined registration submodule (RRS) in a bone-wise manner.

Let $M_{f},M_{t}\in \{0,1{\}}^{H\times W\times D}$ denote binary masks corresponding to the femur and tibia, respectively. These are segmented by a pre-trained UNet (29). Separate rigid transformations are estimated by maximizing mutual information within each bone region:

$$R_{f}=\arg\underset{R}{\max}\;MI(R(I^{sCT}⊙M_{f}),I^{CT}⊙M_{f})$$

$$R_{t}=\arg\underset{R}{\max}\;MI(R(I^{sCT}⊙M_{t}),I^{CT}⊙M_{t})$$

The refined registered MR image is obtained by composing the bone-specific transformations:

$$I^{rrMR}=R_{f}(I^{sCT})⊙M_{f}+R_{t}(I^{sCT})⊙M_{t}$$

This step yields anatomically consistent MR–CT alignment at the bone level, enabling reliable extraction of corresponding patches for downstream regression.

### 3.2 Distillation-learning-based regression module (DLRM)

After establishing accurate anatomical correspondence, paired MR and CT patches can be extracted from the refined registered images. The objective of this module is to construct a CT-based regression model capable of predicting trabecular parameters that are otherwise difficult to recover from low-resolution CT alone.

**Figure 3:**
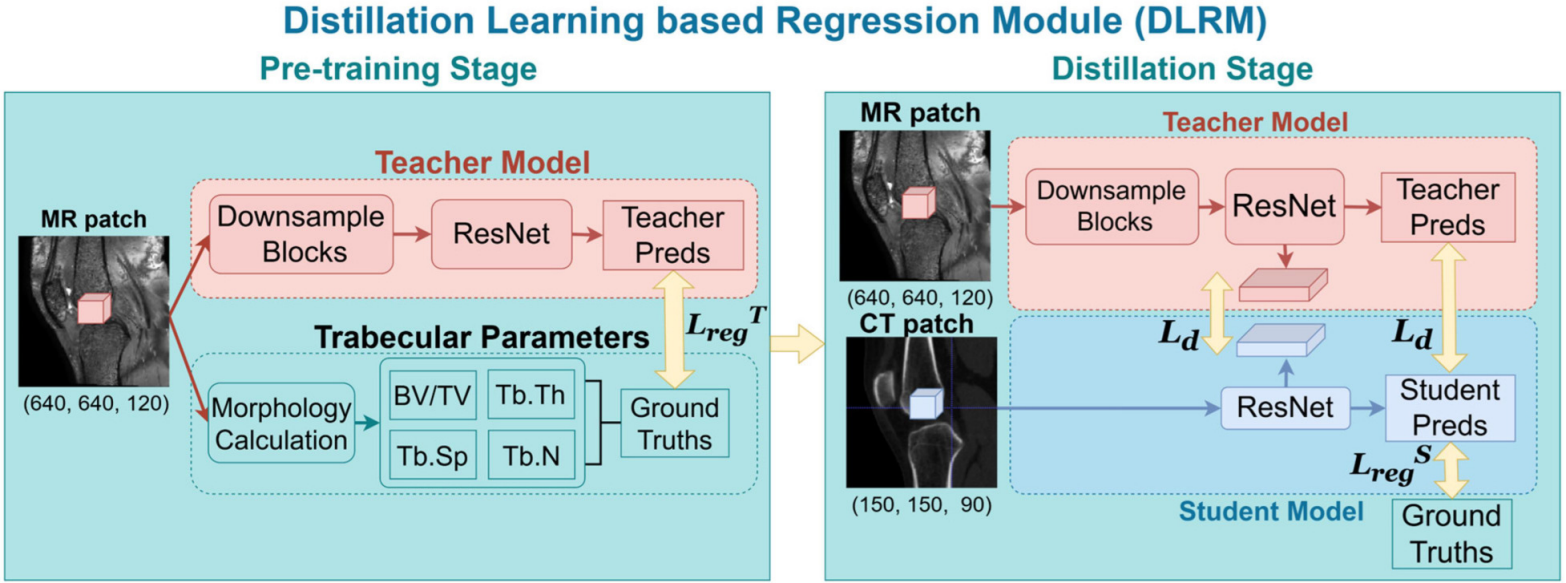
Details of distillation-learning-based regression module (DLRM).

Direct training on whole volumes is infeasible due to limited sample size. Therefore, we adopt a patch-level learning strategy, which substantially increases the number of training samples and allows the model to focus on local trabecular patterns. Patch-level predictions are later aggregated to support patient-level osteoarthritis analysis. To compensate for the limited spatial resolution of CT, as illustrated in Figure 3, we employ a teacher–student distillation framework, in which high-resolution MR patches guide the learning of the CT-based model.

#### 3.2.1 Teacher model pre-training

In the pre-training stage, the trabecular parameters obtained from morphology calculation serve as the ground-truths, and the teacher model *T* is trained to predict these parameters directly from the MR image patches. Let $x_{i}^{MR}$ denote an MR patch and $y_{i}=(\mathrm{BV}/\mathrm{TV},\mathrm{Tb}.\mathrm{Th},\mathrm{Tb}.\mathrm{Sp},\mathrm{Tb}.N)\in R^{4}$ denote the corresponding trabecular parameter ground truths. The teacher prediction is

$${\hat{y}}_{i}^{T}=T(x_{i}^{\mathrm{MR}}),$$

and the teacher is optimized using a regression loss that minimizes the discrepancy between predictions and ground truth parameters:

$$L_{\mathrm{reg}}^{T}=\|{\hat{y}}_{i}^{T}-y_{i}{\|}_{2}^{2}.$$

#### 3.2.2 Distillation-guided student training

The student network S learns to regress trabecular parameters from CT patches $x_{i}^{CT}$. Its prediction is denoted as

$${\hat{y}}_{i}^{S}=S(x_{i}^{CT}).$$

Knowledge distillation is applied at both the prediction and feature levels. The distillation loss is defined as

$$L_{d}=w_{1}|\left|{\hat{y}}_{i}^{S}-{\hat{y}}_{i}^{T}\right|{|}_{2}^{2}\;+\overset{K}{\underset{i=1}{\sum }}\;w_{2,j}\|M_{S,i}^{j}-M_{T,i}^{j}{\|}_{1},$$

where for *i*-th patch $M_{S,i}^{j}$ and $M_{T,i}^{j}$ denote the *j*-th intermediate feature maps of the student and teacher networks, respectively. The prediction-level term encourages the student to approximate the teacher's output, while the feature-level term promotes alignment of internal representations across modalities.

There is also a supervised regression loss $L_{\mathrm{reg}}^{S}$ that penalizes the discrepancy between the student prediction and the MR-derived reference $y_{i}$:

$$L_{\mathrm{reg}}^{S}=\|{\hat{y}}_{i}^{S}-y_{i}{\|}_{2}^{2}.$$

During inference, only the CT-based student model is used. The regressed trabecular parameters are subsequently utilized for knee osteoarthritis analysis.

### 3.3 Patch-level aggregation and patient-level OA classification

The trabecular parameters regressed from individual CT patches characterize local subchondral bone microstructure. However, knee osteoarthritis is a joint-level disease whose manifestation is spatially heterogeneous, which cannot be reliably inferred from isolated local measurements. Therefore, patient-level diagnosis requires integrating trabecular information across multiple anatomical regions while preserving their spatial context.

To this end, we introduce a Transformer-based aggregation module that fuses patch-level trabecular parameters into a global representation for osteoarthritis classification. This proposed design explicitly models inter-patch relationships and enables the network to capture long-range dependencies among spatially distributed microstructural patterns.

#### 3.3.1 Patch-level feature construction

For each subject, the CT-based student model produces a set of patch-level trabecular parameter predictions. Let $\hat{y_{i}}=(\hat{{\mathrm{BV}/\mathrm{TV}}_{i}},\hat{\mathrm{Tb}.Th_{i}},\hat{\mathrm{Tb}.Sp_{i}},\hat{\mathrm{Tb}.N_{i}})\in R^{4}$ denote the predicted trabecular parameters for the *i*-th patch. While these parameters encode local microstructural properties, they do not contain explicit spatial information.

To incorporate anatomical context, each patch is associated with its spatial coordinate $c_{i}\in R^{3}$, defined in the CT image coordinate system. A positional embedding function ϕ(⋅) maps the coordinate into a low-dimensional representation. The final patch descriptor is constructed by concatenating trabecular parameters and positional encoding $e_{i}=\phi (c_{i})$. This design allows the aggregation module to jointly reason about microstructural characteristics and their spatial distribution within the joint.

#### 3.3.2 Transformer-based aggregation

Each patch descriptor $u_{i}=[\hat{y_{i}};e_{i}]$ is first projected into a d-dimensional embedding space using a linear transformation $z_{i}=W_{e}u_{i}+b_{e}$, where $W_{e}$ and $b_{e}$ denote learnable parameters. The resulting sequence $\{z_{i}{\}}_{i=1}^{N}$ is treated as a set of patch tokens and processed by a Transformer encoder consisting of two stacked layers:

$$\{z_{i}^{\left(2\right)}{\}}_{i=1}^{N}=\mathrm{TransformerLayer}(\{z_{i}{\}}_{i=1}^{N}).$$

Within each Transformer layer, self-attention mechanisms allow each patch token to selectively attend to other patches, enabling the model to capture correlations between trabecular patterns at different anatomical locations. This is particularly important for osteoarthritis analysis, where disease-related changes often emerge as coordinated alterations across multiple subregions rather than isolated local abnormalities.

After two Transformer layers, the updated patch embeddings are aggregated into a global representation using average pooling:

$$h=\frac{1}{N}\overset{N}{\underset{i=1}{\sum }}\;z_{i}^{\left(2\right)}.$$

The vector *h* summarizes joint-level trabecular microstructural characteristics and serves as the patient-level feature representation.

#### 3.3.3 OA classification

The aggregated representation *h* is fed into a classification head to predict the osteoarthritis grade of the subject. Specifically, a linear classifier followed by a softmax activation produces a multi-class prediction:

$$\hat{o}=\mathrm{softmax}(W_{o}h+b_{o}),$$

where $\hat{o}$ denotes the predicted probability distribution over OA classes, and $W_{o}$ and $b_{o}$ are learnable parameters. The model is trained using a cross-entropy loss:

$$L_{\mathrm{cls}}=-\overset{C}{\underset{c=1}{\sum }}\;o_{c}^{*}\;\log\hat{o_{c}},$$

where $o_{c}^{*}$ is the ground-truth OA label for *c*-th OA class. By integrating spatially distributed trabecular parameters into a unified representation, this module enables CT-SMA to perform patient-level osteoarthritis classification using only CT data at inference time.

## 4 Experiments and results

To evaluate the superiority of our proposed models, we first introduce experiments settings, and then sequentially compared the performances of different methods on MR-to-CT synthesis, cross-modal registration, trabecular parameter regression, and KOA analysis.

### 4.1 Dataset and preprocessing

**Figure 4:**
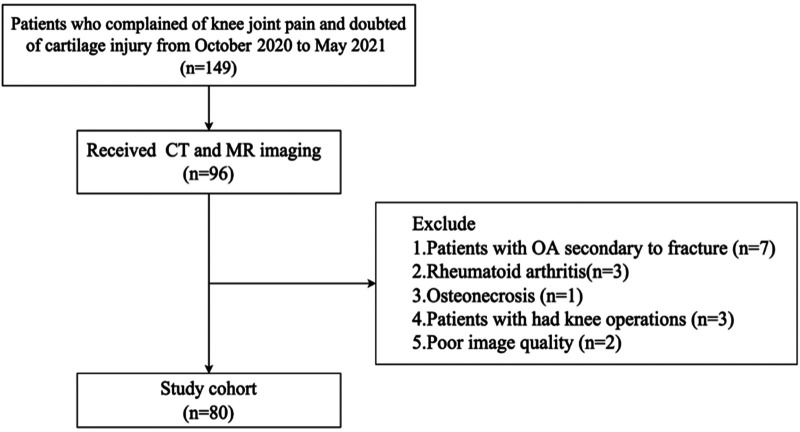
Flowchart illustrating the patient selection process in this study.

This retrospective study was approved by the institutional review board of Shanghai Ninth People's Hospital, and written informed consent was obtained from all participants. A total of 149 consecutive subjects with knee pain and suspected cartilage injury were recruited between October 2020 and May 2021. Among them, 96 subjects underwent both CT and MR-BFFE imaging. The flowchart of patient selection is presented in Figure 4. After excluding cases with secondary osteoarthritis due to fracture, rheumatoid arthritis, osteonecrosis, prior knee surgery, or insufficient image quality, a final cohort of 80 subjects was included in the analysis. The specific demographic information of the selected subjects is listed in Table 1.

**Table 1:** Demography analysis of patients with various stages of OA.

Variables	Total (*n* = 80)	Normal (*n* = 30)	Mild OA (*n* = 25)	Advanced OA (*n* = 25)	*P* value
Ages (Years)	51.3 ± 14.5	38.6 ± 9.6	56.3 ± 9.7	62.4 ± 10.1	< 0.001
Sex					0.58
Male	45 (56.2%)	15 (33.3%)	14 (31.1%)	16 (35.6%)	
Female	35 (43.8%)	15 (42.9%)	11 (31.4%)	9 (25.7%)	
BMI	25.2 ± 3.1	24.6 ± 2.7	24.9 ± 3.6	26.1 ± 2.6	0.16
Knee					0.78
Left knee	41 (51.2%)	14 (34.1%)	13 (31.7%)	14 (34.1%)	
Right knee	39 (48.8%)	16 (41.0%)	12 (30.8%)	11(28.2%)	

BMI, body mass index. Ages and BMI are cited as mean values ± standard deviations.

Subjects were stratified into three groups according to the Kellgren–Lawrence (KL) grading system: normal control (KL = 0, *n* = 30), mild OA (KL = 1–2, *n* = 25), and advanced OA (KL = 3–4, *n* = 25). Knee MR images were further assessed using the MRI Osteoarthritis Knee Score (MOAKS), which evaluates cartilage loss across five anatomical subregions.

For preprocessing, MR volumes were cropped to 640 × 640 × 120 voxels, while CT volumes were cropped to 150 × 150 × 90 voxels to define regions of interest covering the knee joint. For training the distillation-learning-based regression (DLRM) module, CT patches of size 12 × 12 × 12 were sampled from trabecular regions. The corresponding MR patches were extracted at the same anatomical locations and resized to 48 × 48 × 16 to match the resolution discrepancy between modalities. More details on patch sampling is provided in Section 4.5. The comparisons of pre-processed MR and CT in in-plane FOV, resolution and scanner are listed in the Table 2.

**Table 2:** Comparison of FOV, resolution and scanner of MR and CT images.

Imaging parameter	MR image	CT image
In-plane FOV	640 × 640 × 120	150 × 150 × 90
Resolution (mm^3^)	0.234 × 0.234 × 1.500	0.977 × 0.977 × 1.000
Scanner	Philips Healthcare Achieva 3.0TX	Philips Brilliance 64 CT

### 4.2 Implementation and evaluation

All components of the proposed CT-SMA framework were implemented in PyTorch and trained on a single NVIDIA Tesla A100 GPU. Quantitative results are reported as mean ± standard deviation over five-fold cross-validation. All cross-validation splits were performed strictly at the patient level to prevent information leakage. Specifically, all patches derived from a given subject were assigned to the same fold, ensuring that no patches from the same patient appeared in both training, validation and test sets.

The training details of networks in different modules of CT-SMA are listed in Table 3. The details include the dimension, learning rate, optimizer, training epoch, and datasets used for training.In addition to the optimization settings listed in Table 3, regularization strategies were applied to mitigate overfitting. Specifically, dropout (rate = 0.5) was employed in both the DLRM and Transformer aggregation modules, and weight decay of 1 × 10^−^⁴ was applied during training. For the SPSNet module, paired geometric data augmentation, including random flipping, rotation, and spatial shifting, was performed consistently on MR–CT image pairs to preserve anatomical correspondence. Further implementation details and regularization configurations are provided in Supplementary Material A1.

**Table 3:** The training details of networks in our proposed CT-SMA.

Networks	Dimension	Learning rate	Optimizer	Epochs	Dataset
SPSNet	2.5D	0.0002	Adam	100	crMR, CT
UNet	2.5D	0.0001	Adam	50	CT
DLRM	3D	0.0003	Adam	50	MR, CT

UNet is used for segmentation of bone components in refined registration submodule.

The proposed CT-SMA framework performs CT-only inference and does not require MR data or registration at deployment. On a workstation equipped with an Intel Core i5-13600 CPU and an NVIDIA RTX 4090 GPU, the average end-to-end inference time is approximately 2.3 s per subject (GPU inference). Additional computational and deployment details are provided in Supplementary Material A2.

Registration accuracy was evaluated using target registration error (TRE) computed on corresponding anatomical landmarks in MR and CT images. Regression performance was assessed using intraclass correlation coefficients (ICCs) for trabecular parameters, including BV/TV, Tb.Th, Tb.Sp, and Tb.N. For osteoarthritis classification, precision, recall, F1-score, and area under the ROC curve (AUC) with 95% confidence intervals were reported. Statistical significance between methods was assessed using paired t-tests with a 5% significance level.

### 4.3 Results for knee MR-CT registration

#### 4.3.1 MR-to-CT synthesis

**Figure 5:**
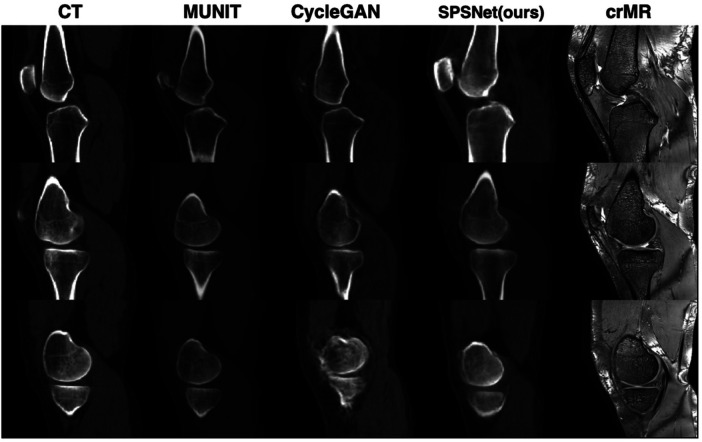
Comparison of crMR-to-sCT synthesis using different methods. Each row displays the synthesis results of a sampled slice from the test dataset. The columns, arranged from left to right, represent CT images, sCT images by MUNIT (30), sCT images by CycleGAN (31), sCT images by our proposed SPSNet and the original crMR images, respectively. crMR = coarsely-registered MR, sCT = synthesized CT.

We first evaluate the performance of MR-to-CT synthesis using SPSNet. As the synthesis submodule aims to perform modality transformation while retaining anatomical structures, we commence by comparing the anatomical similarity between sCT and crMR, and image intensity distribution consistency between sCT and CT. The qualitative synthesis results are shown in Figure 5, which compares our SPSNet with CycleGAN (31) and MUNIT (30). It can be observed that CycleGAN fails to preserve the anatomical structures of crMR, and both MUNIT and CycleGAN result in noticeable distortions in the femur region of sCT. In contrast, our proposed SPSNet preserves more anatomical structure and accomplishes successful modality transformation from crMR.

To further quantify the anatomical structural similarity between sCT and crMR, the TRE of landmarks is tabulated in Table 4. Significantly lower TRE (2.93 ± 1.75 mm) is observed on sCT images generated by our proposed SPSNet than CycleGAN (6.70 ± 4.22 mm) and MUNIT (4.03 ± 2.81 mm).

**Table 4:** The TRE comparison for the MR-to-CT synthesis and the overall registration.

MR-to-CT Synthesis (crMR-sCT)			
Methods	CycleGAN (31)	MUNIT (32)	SPSNet (ours)
TRE/mm	6.70 ± 4.22	4.03 ± 2.81	**2.93** **±** **1.75**
	(95% CI: 5.78–7.62)	(95% CI: 3.41–4.65)	**(95% CI: 2.55–3.31)**
Overall Registration (MR-CT)			
Methods	ANTs (33)	ANTs (33) + SPSNet	ANTs (33) + SPSNet + RRS (ours)
TRE/mm	13.56 ± 8.41	8.06 ± 4.98	**3.67** **±** **2.43**
	(95% CI: 11.72–15.40)	(95% CI: 6.97–9.15)	**(95% CI: 3.14–4.20)**

RRS, Refined registration submodule. TRE values are reported as mean ± standard deviation, with 95% confidence intervals shown in parentheses. In the synthesis process, a lower TRE indicates a smaller spatial gap between the same landmark in the synthesized and real images. For the overall registration, lower TREs indicates better registration results.

Bold values indicate the lowest registration error in each row.

This improvement can be attributed to the design of SPSNet, which explicitly constrains structural consistency during cross-modal synthesis. By preserving the physical locations of anatomical structures, SPSNet effectively reduces the multi-modal registration problem to a mono-modal registration between synthesized CT and real CT, thereby simplifying subsequent alignment and improving robustness.

#### 4.3.2 Overall MR-to-CT registration

Building upon the synthesized CT images, we evaluate the overall MR-to-CT registration performance, which aims to align the two modalities under simultaneous differences in resolution, intensity distribution, and knee-bent angles. By converting the original multi-modal registration problem into a mono-modal registration between synthesized CT and real CT, the synthesis submodule provides a more favorable initialization for subsequent alignment.

**Figure 6:**
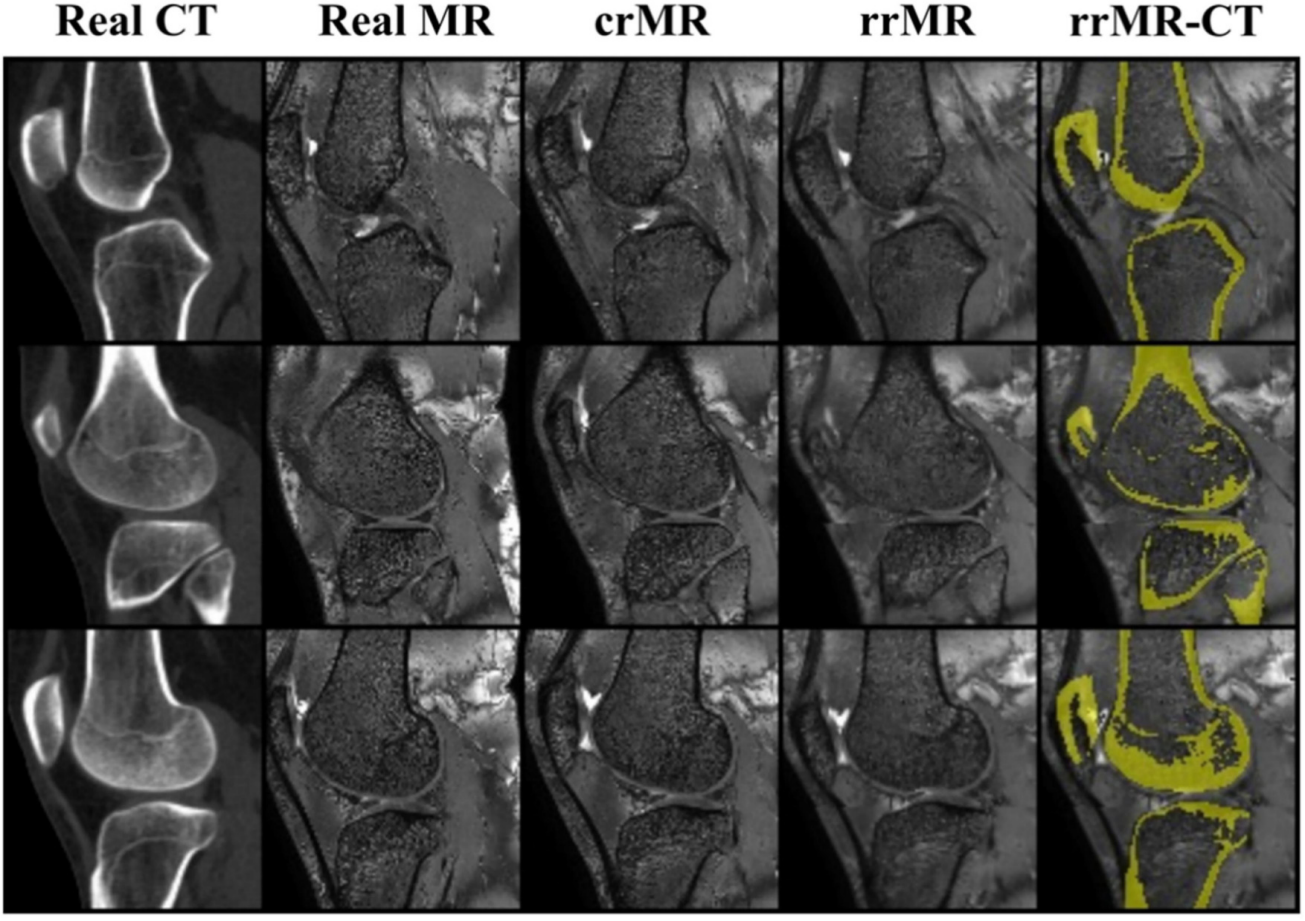
Comparisons of different registration methods. Each row shows the synthesis results of a sampled slice from the test dataset. From left to right, the columns are sequentially real MR images, real CT images, coarsely-registered MR (crMR) images by ANTs (rigid only) (32), refined-registered MR images (rrMR), and the visualization of registration performance between rrMR and real CT. In the visualization images, the high signals on the registered CT images are colored yellow and overlaid onto the corresponding rrMR images. The visualization shows a satisfied structural alignment situation of rrMR and CT images.

Qualitative comparisons of the overall registration are shown in Figure 6. While the tibia region is well registered in crMR, unsatisfactory registration is observed in the femur due to the angle differences between the femur and tibia. The proposed refined registration submodule (RRS) addresses this issue by separately registering the femur and tibia regions, enabling more accurate compensation for relative angular discrepancies and yielding improved alignment in the refined registered MR (rrMR) images.

Quantitative ablation results using target registration error (TRE) are also summarized in Table 4. Compared to the coarse registration by ANTs (32), SPSNet achieves modality transformation and mitigates misalignment caused by intensity distribution differences during registration, yielding a lower TRE of 8.06 ± 4.98 mm (vs. 13.56 ± 8.41 mm, P<.001). Furthermore, by explicitly modeling knee-bent angle variations through region-wise rigid registration, the refined registration submodule further reduces TRE to 3.67 ± 2.43 mm (*P* < .001), demonstrating its effectiveness in handling joint-specific geometric inconsistencies.

### 4.4 Results for distillation-learning-based regression (DLRM)

The regression module aims to estimate trabecular microstructural parameters from CT image patches. Quantitative results, summarized in Table 5, report the intraclass correlation coefficients (ICCs) between the regressed parameters and the corresponding ground truth derived from high-resolution MR images.

**Table 5:** ICC comparison of trabecular parameter regression results.

Trabecular parameter	Radiomic-feature-based model	CNN regression model	Distillation-learning-based CNN regression model
BV/TV	0.548 ± 0.011	0.670 ± 0.053	0.804 ± 0.037
Tb.Th	0.407 ± 0.032	0.488 ± 0.038	0.773 ± 0.042
Tb.Sp	0.356 ± 0.017	0.523 ± 0.054	0.711 ± 0.063
Tb.N	0.326 ± 0.036	0.502 ± 0.085	0.622 ± 0.133
Overall	0.409 ± 0.026	0.545 ± 0.059	0.742 ± 0.046

The compared regression methods include the radiomic-feature-based model, CNN regression model, and the proposed distillation-learning-based CNN regression model. The trabecular parameters that were regressed are BV/TV, Tb.Th, Tb.Sp, and Tb.N. BV/TV, bone volume/total volume; Tb.Th, trabecular thickness; Tb.Sp, trabecular separation; Tb.N, trabecular number.

Compared with radiomic-feature-based regression (ICC: 0.409 ± 0.024), CNN-based models achieve notably higher ICCs (0.545 ± 0.067), indicating the advantage of learning hierarchical representations directly from image patches. However, directly applying mono-modal CNN regression to CT remains challenging due to the limited spatial resolution of conventional CT and the substantial appearance discrepancy between CT and MR, from which the ground-truth parameters are computed.

**Figure 7:**
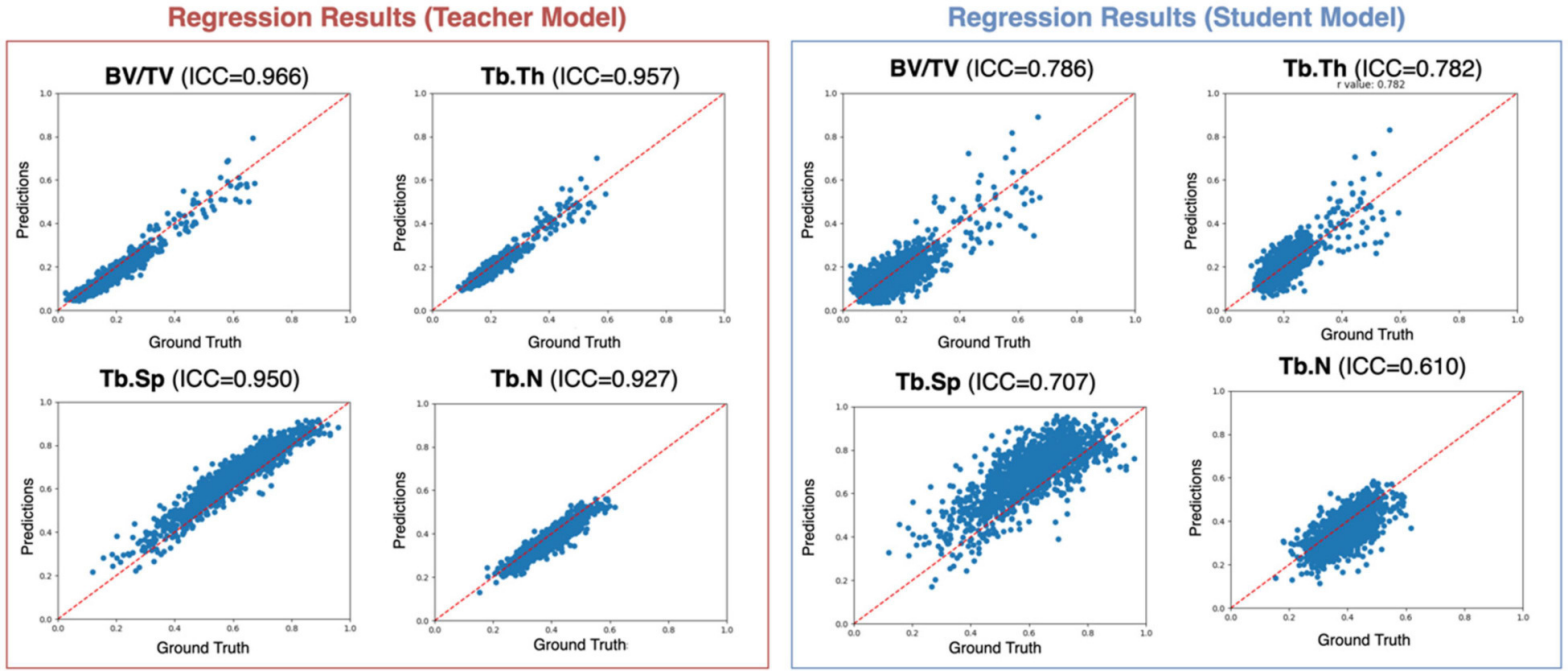
Scatter plots of trabecular parameter regression results. Both the regression results from the teacher (left) and student (right) models with four plots representing BV/TV, Tb.Th, Tb.Sp, and Tb.N are presented. Each blue point represents a regression result from a single CT patch in the trabecular section of the knee of one patient. BV/TV, bone volume/total volume; Tb.Th, trabecular thickness; Tb.Sp, trabecular separation; Tb.N, trabecular number.

By incorporating the proposed distillation learning strategy, the CT-based student model is guided by a well pre-trained MR-based teacher model, effectively transferring microstructural knowledge encoded in high-resolution MR patches. As shown in Figure 7, this cross-modal supervision substantially improves regression performance, yielding a mean ICC of 0.742 ± 0.046 (*P* < .001). Importantly, although MR information is exploited during training, the resulting model operates exclusively on CT data at inference time, demonstrating the feasibility of CT-based subchondral bone microstructural analysis despite pronounced modality and resolution differences.

To evaluate sensitivity to residual MR–CT misalignment, we performed a controlled perturbation analysis by introducing synthetic translation errors (1–15 mm) into MR patch sampling coordinates. Regression agreement (ICC) showed minimal degradation under small perturbations (1–2 mm), moderate decline at 5 mm, and substantial deterioration when misalignment approached or exceeded the patch extent (10–15 mm), indicating graceful degradation under anatomically plausible residual errors (see Supplementary Section B.1).

### 4.5 KOA analysis based on regressed trabecular parameters

Following patch-level trabecular parameter regression, we further evaluated the proposed CT-SMA framework in patient-level knee osteoarthritis (KOA) analysis. Regressed trabecular parameters obtained from different regression methods were adopted as input features for two clinically relevant tasks: KL-grade-based KOA classification and MOAKS score prediction.

The trabecular regions of the femur and tibia were first automatically segmented using a pre-trained 3D U-Net model following (33). The resulting bone masks were subsequently reviewed and manually refined by a radiologist with 15 years of experience to ensure anatomical accuracy. Patch sampling for the DLRM module was restricted to these validated trabecular regions. Consistent with prior work (34), each trabecular region of the femur and tibia was partitioned into K = 100 anatomically coherent subregions using maskSLIC (35). For each subregion, trabecular parameters regressed from the central patch were extracted and aggregated as patient-level descriptors. To avoid excessive redundancy, the patch size for CT is chosen at 12 × 12 × 12 (approximately 11.74 × 11.74 × 12 mm^3^), which ensures the intersection-over-union (IoU) between any two sampled CT patches remained below 0.5 within a subject across the dataset. To investigate the impact of different aggregation strategies, we compared three types of patient-level models: a Multi-Layer Perceptron (MLP) (36), a CNN-based aggregation model, and a Transformer-based aggregation model.

The MLP serves as a lightweight baseline commonly adopted under limited patient-level supervision. The CNN-based model introduces local interaction among subregions but remains constrained by fixed receptive fields. In contrast, the Transformer-based model explicitly models long-range dependencies among spatially distributed trabecular subregions through self-attention, enabling more effective reasoning over global microstructural patterns within the joint.

**Figure 8:**
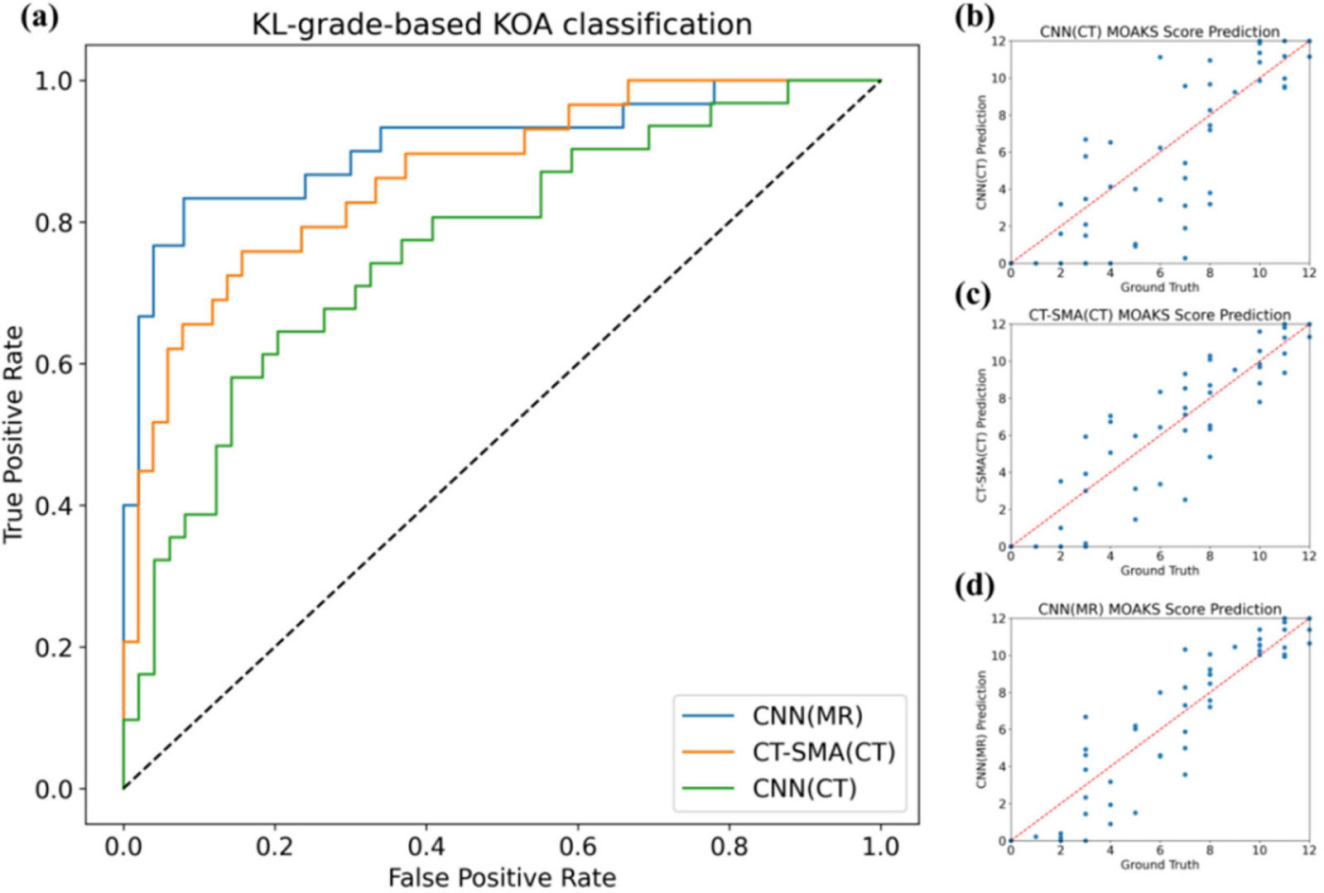
Results of KOA analysis based on regressed trabecular parameters. **(a)** presents the Receiver Operating Characteristic (ROC) curve of KL-grade-based KOA classification based on regressed trabecular parameters. The green line corresponds to the performance of a CNN on CT images, the orange line indicates the performance using CT images with the proposed CT-SMA method, and the blue line represents the performance using MR images as a reference. **(b**–**d)** are scatter plots of cartilage score predictions based on regressed trabecular bone parameters. **(b)** shows predictions from CT images using a CNN, **(c)** illustrates predictions from CT images using the proposed CT-SMA method, and **(d)** displays predictions from MR images using a CNN for reference.

Quantitative results for OA vs. non-OA classification are summarized in Table 6, with ROC curves and scatter plots illustrated in Figure 8. For KL-grade-based (OA vs. non-OA) classification, CT-SMA achieves a significantly higher AUC compared to radiomic-based methods (*P* < .001) and vanilla CNN-based regression methods (*P* < .001). This performance gain can be attributed to the improved quality of patch-level trabecular features produced by the distillation-guided regression module, which captures microstructural patterns that are more discriminative for disease staging. To further assess staging capability, we additionally evaluated the model for OA severity stratification (Mild OA: KL = 1–2 vs. Advanced OA: KL = 3–4) using the existing predictions. The corresponding AUC and F1-score results are provided in the Supplementary Materials Section B.3.

**Table 6:** The statistical analysis of KL-grade-based KOA classification and MOAKS score prediction using trabecular parameters from different regression methods.

Methods	Metrics	Radiomic-based (CT)	CNN (CT)	CT-SMA (CT)	CNN (MR)
KL-grade-based KOA classification	Precision	0.689 ± 0.078	0.745 ± 0.041	**0.863** ± **0.053**	0.887 ± 0.061
	Recall	0.773 ± 0.045	0.794 ± 0.047	**0.903** ± **0.068**	0.935 ± 0.037
	F1 score	0.738 ± 0.057	0.763 ± 0.040	**0.879** ± **0.056**	0.910 ± 0.044
	AUC	0.742 ± 0.032	0.778 ± 0.044	**0.883** ± **0.029**	0.914 ± 0.048
MOAKS score prediction	Mean Average Error	1.438 ± 0.153	1.059 ± 0.072	**0.893** ± **0.041**	0.876 ± 0.038
	R-Square	0.723 ± 0.129	0.790 ± 0.064	**0.881** ± **0.037**	0.903 ± 0.030

The classification is implemented using regressed trabecular parameters on image patches as features. Here we compare different methods to obtain trabecular parameters and the employed aggregation for different methods is Transformer layers. We compare radiomic-based method, CNN-based method and our proposed CT-SMA on CT images. Results of CNN-based method on MR images are provided in the right column for references.

Bold values indicate the best performance among the CT-based methods.

Moreover, we compare the methods to aggregate patch-level trabecular parameters for OA analysis tasks as ablation in Table 7. Across both KL-grade-based classification and MOAKS score prediction tasks, a consistent performance ranking is observed among aggregation strategies, with Transformer-based aggregation outperforming CNN-based aggregation, which in turn surpasses the MLP baseline. This trend indicates that modeling global inter-subregion relationships is critical for accurate KOA assessment, as disease-related trabecular alterations often manifest as coordinated changes across multiple anatomical locations rather than isolated local abnormalities. These results further demonstrate that, beyond accurate patch-level regression, the choice of patient-level aggregation model plays a decisive role in translating microstructural predictions into clinically meaningful KOA outcomes.

**Table 7:** The statistical analysis of KL-grade-based KOA classification and MOAKS score prediction using different aggregation methods for patch-level trabecular parameters.

Methods	Metrics	MLP(CT)	CNN (CT)	Transformers (CT)	Transformers (MR)
KL-grade-based KOA classification	Precision	0.843 ± 0.058	0.846 ± 0.051	**0.863** ± **0.053**	0.887 ± 0.061
	Recall	0.836 ± 0.074	0.899 ± 0.065	**0.903** ± **0.068**	0.935 ± 0.037
	F1 score	0.844 ± 0.067	0.868 ± 0.050	**0.879** ± **0.056**	0.910 ± 0.044
	AUC	0.742 ± 0.032	0.872 ± 0.033	**0.883** ± **0.029**	0.914 ± 0.048
MOAKS score prediction	Mean Average Error	1.076 ± 0.146	0.945 ± 0.052	**0.893** ± **0.041**	0.876 ± 0.038
	R-Square	0.837 ± 0.062	0.866 ± 0.044	**0.881** ± **0.037**	0.903 ± 0.030

The classification is implemented using regressed trabecular parameters on image patches as features. Here we compare different aggregation methods to employ regressed trabecular parameters. Our CT-SMA employs the proposed Transformers layer for aggregation. The first four rows are results of KL-grade-based KOA classification.

Bold values indicate the best performance among the CT-based methods.

Although a performance gap remains between CT-SMA and MR-based analysis (*P* = .016), likely due to the inevitable information loss caused by the resolution discrepancy between CT and high-resolution MR, CT-SMA attains an AUC of 0.883 (95% CI, 0.854–0.912). This result highlights the strong potential of CT-based KOA diagnosis, especially considering the lower cost and broader availability of CT imaging in clinical practice. Furthermore, for MOAKS score prediction, CT-SMA achieves an R-square value of 0.881 ± 0.037, demonstrating performance comparable to MR-based methods (*P* = 0.143).

## 5 Conclusions and discussions

In this paper, we propose a distillation-learning-based method namely CT-SMA for trabecular parameter regression, and investigate the feasibility of CT-based subchondral-bone-microstructural-analysis. The proposed CT-SMA framework is composed of three tightly integrated modules that jointly address cross-modal correspondence construction, microstructural knowledge transfer, and patient-level osteoarthritis modeling. Specifically, CT-SMA consists of (1) a synthesis-based MR–CT registration module for establishing anatomically consistent cross-modal patch correspondences, (2) a distillation-learning-based regression module (DLRM) that transfers high-resolution trabecular knowledge from MR to CT, and (3) a Transformer-based aggregation module that integrates spatially distributed trabecular parameters for patient-level OA prediction.

Experimental results substantiate the functional role of each module within CT-SMA. The synthesis-based registration module enables stable and anatomically meaningful MR–CT alignment, which is essential for constructing reliable cross-modal patch pairs and avoiding error propagation in subsequent learning stages. Built upon these correspondences, the DLRM consistently improves the agreement between CT-predicted trabecular parameters and MR-derived references, as reflected by substantially increased intraclass correlation coefficients. These results indicate that, despite the limited spatial resolution of clinical CT, the proposed framework can recover microstructural patterns with sufficient fidelity for quantitative analysis.

Furthermore, downstream osteoarthritis prediction experiments indicate that CT-based trabecular parameters regressed by CT-SMA preserve sufficient disease-discriminative information, yielding patient-level performance comparable to MR-based analysis while requiring only CT at inference. This demonstrates that the transferred microstructural knowledge is both quantitatively consistent and effective for joint-level OA modeling, supporting the applicability of CT-SMA in large-scale population studies.

In addition, we evaluated the computational efficiency of the deployed model. On a standard GPU workstation, the average end-to-end inference time was approximately 2–3 s per subject, and the model size remains within a lightweight deployment range. These results suggest that CT-SMA can be integrated into routine CT-based analysis pipelines without substantial computational burden. Although prospective validation and full integration into clinical workflows (e.g., PACS systems or inter-reader studies) were beyond the scope of this retrospective study, the computational feasibility observed here supports the potential for future real-world deployment and workflow integration.

More broadly, the present study demonstrates that CT-based assessment of subchondral microstructure can be reformulated as a structured cross-modal learning problem. By constructing anatomically aligned correspondences and transferring morphometric knowledge from MR to CT at the patch level, CT-SMA bridges the gap between routine CT accessibility and high-resolution structural characterization. This system-level integration enables CT to serve not merely as a coarse anatomical modality, but as a source of quantitatively meaningful structural biomarkers for osteoarthritis modeling.

The limitations of this study and potential remedies are listed as follows: First, the cohort was derived from a single institution with a consistent scanner configuration and a relatively homogeneous population, which may introduce acquisition-specific and selection bias and limit generalizability. Although the cohort size (*n* = 80) is comparable to several prior imaging studies of trabecular microstructure (2, 3, 10), a larger cohort would provide greater statistical power for subgroup analysis and more robust estimation of model performance. Scanner-dependent imaging characteristics and population-specific baseline bone properties may influence structural feature distributions under domain shifts. Future multi-center validation across diverse scanners and populations will be necessary to assess robustness and improve external generalizability. Second, although the selected trabecular morphometric parameters (BV/TV, Tb.Th, Tb.Sp, and Tb.N) are widely adopted structural descriptors associated with OA severity, they capture only part of the complex pathological processes involved in KOA. Additional topology-based measures (37, 38) or complementary clinical and imaging variables (39) may further enhance phenotyping. Future extensions may incorporate broader structural or multimodal descriptors to provide more comprehensive disease characterization. Finally, MR-derived morphometric parameters serve as established *in vivo* structural surrogates but do not represent direct histological or micro-CT ground truth. The present study therefore evaluates CT-SMA relative to MR-equivalent biomarkers rather than micron-scale trabecular architecture. Dedicated *ex vivo* validation, biomechanical correlation studies, or longitudinal outcome-based assessments may further strengthen biological interpretability.

## Data Availability

The original contributions presented in the study are included in the article/Supplementary Material, further inquiries can be directed to the corresponding author.
