# Supplementary material for: CT-based subchondral bone microstructural analysis in knee osteoarthritis via MR-guided distillation learning
Source: Front Radiol. 2026 Apr 10;6:1798348. doi: 10.3389/fradi.2026.1798348 (PMC13106955; doi:10.3389/fradi.2026.1798348)
Supplement: Supplementary file 1 [file Datasheet1.docx]

**Supplementary Materials**

**A1. Detailed Implementation and Regularization Settings**

All networks were implemented in PyTorch. Patch size is described in the main manuscript. For each subject, K=100 subregions were sampled for regression and aggregation.

SPSNet was trained in a 2.5D manner with a batch size of 16 using the Adam optimizer. Paired geometric data augmentation was applied to both MR and CT inputs, including random flipping, rotation, and spatial shifting, to preserve anatomical correspondence during synthesis-guided registration.

The distillation-learning regression module (DLRM) was implemented in 3D with a batch size of 32 and optimized using Adam. Dropout with a rate of 0.5 was applied after fully connected layers to mitigate overfitting. Weight decay was set to 1×10^−4^.

The patient-level aggregation module consists of a lightweight Transformer encoder with 2 layers, embedding dimension 384, and 12 attention heads. The batch size for Transformer training was 4. Dropout (rate = 0.5) was applied after fully connected layers within the aggregation network. Weight decay of 1×10−4 was also used during optimization.

**A2. Computational Footprint and Deployment Details**

The computational environment for training and inference consisted of a workstation equipped with an Intel Core i5-13600 CPU and an NVIDIA RTX 4090 GPU. All reported inference times correspond to GPU-based inference.

At deployment, CT-SMA operates using CT-only input and does not require MR data or the registration module. The inference pipeline consists of patch-level regression using the trained ResNet-50-based DLRM followed by patient-level aggregation through a lightweight two-layer Transformer encoder.

The average end-to-end inference time was approximately 2.3 seconds per subject under GPU inference. The ResNet-50 backbone occupies approximately 97 MB, and the two-layer Transformer aggregation module occupies approximately 20 MB, resulting in a total model size of approximately 120 MB.

No model pruning, quantization, or compression techniques were applied in the current implementation. Future work will explore model compression strategies to further optimize deployment in resource-constrained environments.

**A3：Artifact robustness discussion for deployment**

In routine clinical environments, CT images may be affected by artifacts such as metal streak artifacts from knee implants, motion-related degradation, or reconstruction noise. The retrospective cohort used in this study primarily consisted of diagnostic-quality scans without extensive post-surgical hardware, and a dedicated artifact-focused evaluation was not performed

CT-SMA performs patch-level regression without explicit trabecular segmentation and aggregates predictions across multiple subregions using a Transformer encoder. This design may partially mitigate the influence of localized intensity distortions; however, substantial artifacts could still affect regression accuracy by altering local attenuation patterns.

Future work will include systematic evaluation on artifact-enriched datasets and investigation of artifact-aware strategies, such as simulation-based augmentation or preprocessing with metal artifact reduction techniques, to further improve robustness for real-world deployment.

**A.4 Sensitivity to Residual MR–CT Misalignment**

To investigate the robustness of distillation-based regression to residual registration errors, we conducted a controlled perturbation analysis. Starting from the refined MR–CT alignment, synthetic residual misalignments were introduced by translating MR patch sampling centers in physical space by random vectors with magnitudes of 1, 2, 5 and 10 mm, while keeping CT patch locations fixed.

The CT patch size was 12 × 12 × 12 voxels (approximately 12 mm in each spatial dimension). Therefore, perturbations approaching or exceeding this spatial extent substantially disrupt anatomical correspondence between paired patches.

As shown in Table S1, regression performance was quantified using the intraclass correlation coefficient (ICC) between predicted and MR-derived trabecular parameters. As shown in Table S1, ICC values remained largely stable under small perturbations (1–2 mm), showed moderate degradation at 5 mm, and declined markedly at 10 mm.

Specifically, within a perturbation range of approximately 2 mm, regression performance remained relatively stable, indicating that CT-SMA is robust to small residual alignment errors that are within clinically plausible registration accuracy. At 5 mm misalignment, performance degradation became more evident; however, partial spatial overlap between corresponding CT and MR patches was still preserved, allowing distillation supervision to retain some structural guidance despite increased noise.

When misalignment reached 10 mm, which approaches the spatial extent of the 12 mm CT patch, anatomical correspondence between paired patches was substantially disrupted. Under this condition, MR-derived supervision no longer reflected the true local CT structural context, and distillation signals became inconsistent. As a result, regression performance declined markedly, approaching or falling below the CT-only baseline without distillation. These findings indicate that cross-modal distillation is effective under anatomically plausible alignment but becomes unreliable when correspondence is severely violated.

These findings indicate that CT-SMA exhibits graceful degradation under small residual misalignment but relies on anatomically plausible patch correspondence for effective cross-modal distillation.

**Table S1**: Intraclass Correlation Coefficient (ICC) Under Controlled Residual MR–CT Misalignment.

| Misalignment /mm | BV/TV | Tb.Th | Tb.Sp | Tb.N | Mean ICC |
| --- | --- | --- | --- | --- | --- |
| 0 | 0.804 ± 0.037 | 0.773 ± 0.042 | 0.711 ± 0.063 | 0.622 ± 0.133 | 0.742 ± 0.046 |
| 1 | 0.793 ± 0.045 | 0.776 ± 0.056 | 0.713 ± 0.058 | 0.616 ± 0.149 | 0.725 ± 0.077 |
| 2 | 0.789 ± 0.048 | 0.748 ± 0.049 | 0.674 ± 0.125 | 0.620 ± 0.139 | 0.708 ± 0.090 |
| 5 | 0.735 ± 0.134 | 0.636 ± 0.183 | 0.593 ± 0.202 | 0.557 ± 0.243 | 0.630 ± 0.191 |
| 10 | 0.597 ± 0.186 | 0.394 ± 0.191 | 0.482 ± 0.293 | 0.396 ± 0.215 | 0.467 ± 0.221 |
| w.o. distillation | 0.670 ± 0.053 | 0.488 ± 0.038 | 0.523 ± 0.054 | 0.502 ± 0.085 | 0.545 ± 0.059 |

**Note.** — Residual misalignment was introduced by applying synthetic random translations to MR patch sampling centers in physical space (mm), while CT patch locations were kept fixed. CT patch size was 12 × 12 × 12 voxels (approximately 12 mm in each spatial dimension). The row “w.o. distillation” denotes the CT-only regression baseline without cross-modal supervision. Mean ICC represents the average of ICC values across the four trabecular parameters.

**B.1 Calibration Analysis and underperforming patterns**

Reliability diagrams for KL-grade-based KOA classification are shown in Figure S2. Overall, all models demonstrate reasonable agreement between predicted and observed probabilities.

In the low-to-intermediate probability range, corresponding to clinically mild or borderline cases, the CT-only model tends to underestimate the observed event rate. In contrast, CT-SMA shows improved alignment with the diagonal in this range, suggesting more stable probability estimation for mild OA cases. At higher probability ranges, calibration performance across models appears comparable.


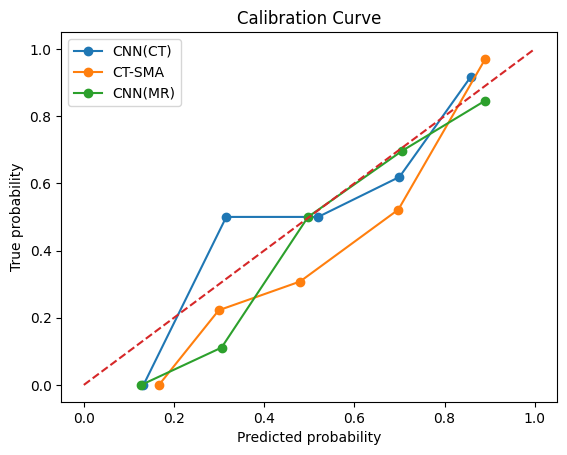


Figure S2. Calibration curves for KL-grade-based KOA classification. Calibration curves were constructed using predicted probabilities from five-fold cross-validation. Predicted probabilities were grouped into equal-width bins, and the observed event rate within each bin was computed. The dashed diagonal line represents perfect calibration.

Compared with the MR-based model, CT-SMA shows slight underperformance in certain borderline cases, particularly in subjects with mild structural alterations. In these cases, CT-SMA tends to assign moderately higher predicted probabilities of OA, occasionally leading to overestimation relative to MR-based assessment. This behavior likely reflects the model’s increased sensitivity to subtle structural signatures learned through distillation. While such shifts may introduce additional false positives in borderline normal cases, they may also reduce the risk of missing early OA, highlighting the inherent trade-off between sensitivity and specificity in clinically ambiguous scenarios.

**B.2 Failure Case Study**

To improve transparency, we present a representative case in which CT-SMA overestimates OA compared with MR-based analysis. In this subject, the ground-truth KL grade was non-OA, and the MR-based model produced a correct negative prediction, whereas CT-SMA predicted OA.

| Method | BV/TV | Tb.Th /mm | Tb.Sp /mm | Tb.N /mm^-1^ |
| --- | --- | --- | --- | --- |
| CT-SMA | 0.136 | 0.172 | 0.524 | 0.193 |
| MR-based | 0.137 | 0.163 | 0.486 | 0.185 |

Quantitative comparison of trabecular parameter regression reveals that the primary deviation originates from Tb.Sp estimation. While BV/TV, Tb.Th, and Tb.N remain relatively consistent with MR-derived references, Tb.Sp is overestimated in this case. Given that Tb.Sp reflects trabecular separation and is sensitive to subtle structural irregularities, small estimation errors may disproportionately influence aggregated OA risk prediction. These results suggest that Tb.Sp estimation under routine CT resolution remains relatively challenging and may contribute to occasional overestimation in mild or normal subjects.

**B.3 OA Severity Stratification Analysis**

To further evaluate the clinical relevance of CT-SMA in OA staging, we conducted an additional severity stratification analysis focusing on OA subjects only. Specifically, OA cases were divided into Mild OA (KL=1–2) and Advanced OA (KL=3–4) according to the Kellgren–Lawrence grading system.

Using the predictions generated by the trained model, we evaluated the ability of CT-SMA to distinguish Mild from Advanced OA. The corresponding ROC curve and performance metrics are summarized in Table S3. The results indicate that CT-SMA retains discriminative capability in differentiating disease severity stages, suggesting that the distilled trabecular structural descriptors capture meaningful progression-related microstructural changes beyond binary OA detection.

**Table S3**: The statistical analysis of KL-grade-based KOA stratification using trabecular parameters from different regression methods.

| Methods | Metrics | Radiomic-based (CT) | CNN (CT) | CT-SMA (CT) | CNN (MR) |
| --- | --- | --- | --- | --- | --- |
| KL-grade-based KOA **Stratification** | F1 score | 0.629±0.083 | 0.736 ± 0.069 | **0.775 ± 0.056** | 0.791 ± 0.047 |
|  | AUC | 0.614±0.076 | 0.723 ± 0.075 | **0.764 ± 0.048** | 0.787 ± 0.054 |

**Note. —**The classification is implemented using regressed trabecular parameters on image patches as features. Here the task is to classify OA cases into mild OA and advanced OA groups.
